# Prehospital whole-blood transfusion in two countries: comparison of patient characteristics in Sweden and the Northeastern United States

**DOI:** 10.1007/s00068-025-03037-9

**Published:** 2026-01-13

**Authors:** Denise Backstrom, Ragnar Henningsson, Norbert Lubenow, Agneta Wikman, Michael Patrick McCartin, Ira J. Blumen, Jeremy F. Norman, David W. Schoenfeld, Stephen H. Thomas

**Affiliations:** 1 Department of Biomedical and Clinical Sciences, Linköping University, Linköping, Sweden; 2 Department of Leadership and Command & Control, Swedish Defense University, Karlstad, Sweden; 3 Department of Anaesthesiology & Intensive Care Central Hospital Karlstad, Region Värmland, Sweden; 4 Department of Clinical Immunology and Transfusion Medicine, Department of Immunology, Genetics and Pathology, Uppsala University Hospital, Uppsala University, Uppsala, Uppsala, Sweden; 5 Department of Clinical Immunology and Transfusion Medicine, Division of Center for Hematology and Regenerative Medicine, Department of Medicine, Karolinska University Hospital, Karolinska Institutet, Stockholm, Sweden; 6 Section of Emergency Medicine & University of Chicago Aeromedical Network, University of Chicago, Chicago, IL USA; 7 Air Methods LLC, Greenwood Village, CO USA; 8 Department of Emergency Medicine, Beth Israel Deaconess Medical Center, Harvard Medical School, Boston, MA USA; 9 Blizard Institute for Neuroscience, Surgery, & Trauma, Barts & The London School of Medicine, London, UK

**Keywords:** Prehospital whole blood, Emergency medical services, Whole blood transfusion, Trauma resuscitation, Sweden, United states, Helicopter Emergency Medical Services (HEMS), Blood product administration, Prehospital care, Alloimmunization

## Abstract

**Background:**

Prehospital transfusion of whole blood (WB) is being adopted in civilian Emergency Medical Services (EMS) systems, but the characteristics of patients receiving WB may differ by region. We aimed to compare prehospital WB transfusion programs in Sweden and in the Northeastern United States, focusing on patient demographics and transfusion details.

**Methods:**

We conducted a retrospective observational study of EMS-initiated WB transfusions in three regions in Sweden (2020–2024) and in multiple EMS programs in the Northeastern United States (2024). We compared patient age, sex (with female patients categorized as of childbearing potential (FCP) if ≤ 50 years), indication for transfusion (trauma vs. non-trauma), and prehospital WB transfusion practice (number of units initiated/completed and RhD type).

**Results:**

A total of 196 patients received prehospital WB (85 in Sweden, 111 in U.S.). Sweden’s WB recipients were younger (median age ~ 41 vs. 59 years) and more often trauma patients (90.6% vs. 72.1%) compared to the U.S. cohort. The proportion of female patients was similar, but Sweden had a higher percentage of FCPs among WB recipients (24% vs. 8%). Swedish EMS were more likely to transfuse multiple units before hospital arrival. Practice differed in RhD type: 97% of U.S. patients received RhD-positive WB, whereas 91% of Swedish patients received RhD-negative WB. Consequently, all 9 FCPs in the U.S. group received at least one Rh-positive unit, compared to 1 of 18 FCPs in Sweden.

**Conclusion:**

Significant differences exist between Sweden and the Northeastern U.S. in prehospital WB transfusion demographics and practices. These findings highlight that one should not assume similar patient profiles or transfusion strategies across different countries’ EMS WB programs.

**Supplementary Information:**

The online version contains supplementary material available at 10.1007/s00068-025-03037-9.

## Background

Prehospital transfusions initiated by Emergency Medical Services (EMS) providers may be lifesaving[1]. EMS transfusions in the civilian setting often include whole blood (WB). Compared to component transfusion, WB administration may offer benefits[2], and WB use for emergency trauma transfusion has been conditionally endorsed by a recent USA-based panel[3].

However, evidence of prehospital transfusion benefit on mortality is not definitive and prehospital transfusion programs are resource-intensive (requiring blood bank support, staff training, blood products and delivery systems). Existing models of prehospital transfusion require input data (e.g., demographics, mortality benefit, alloimmunization risk) that can vary by setting.

The purpose of our study was to assess the WB prehospital transfusion populations of two areas – Sweden and the USA Northeast – both highly developed EMS systems. We aimed to determine whether the population receiving prehospital WB differed in age, sex (with female patients categorized as of childbearing potential (FCP) if ≤ 50 years), transfusion indication (trauma vs. non-trauma), and also to compare observed transfusion practices (number of units initiated/completed) between the two countries.

## Methods

### Design

This was an observational retrospective study of prehospital EMS data. Methodology followed guidelines of *Strengthening Reporting of Observational Studies in Epidemiology* (STROBE); the STROBE checklist appears in the Supplement [4]. 

### Study setting and data sources

**Fig. 1:**
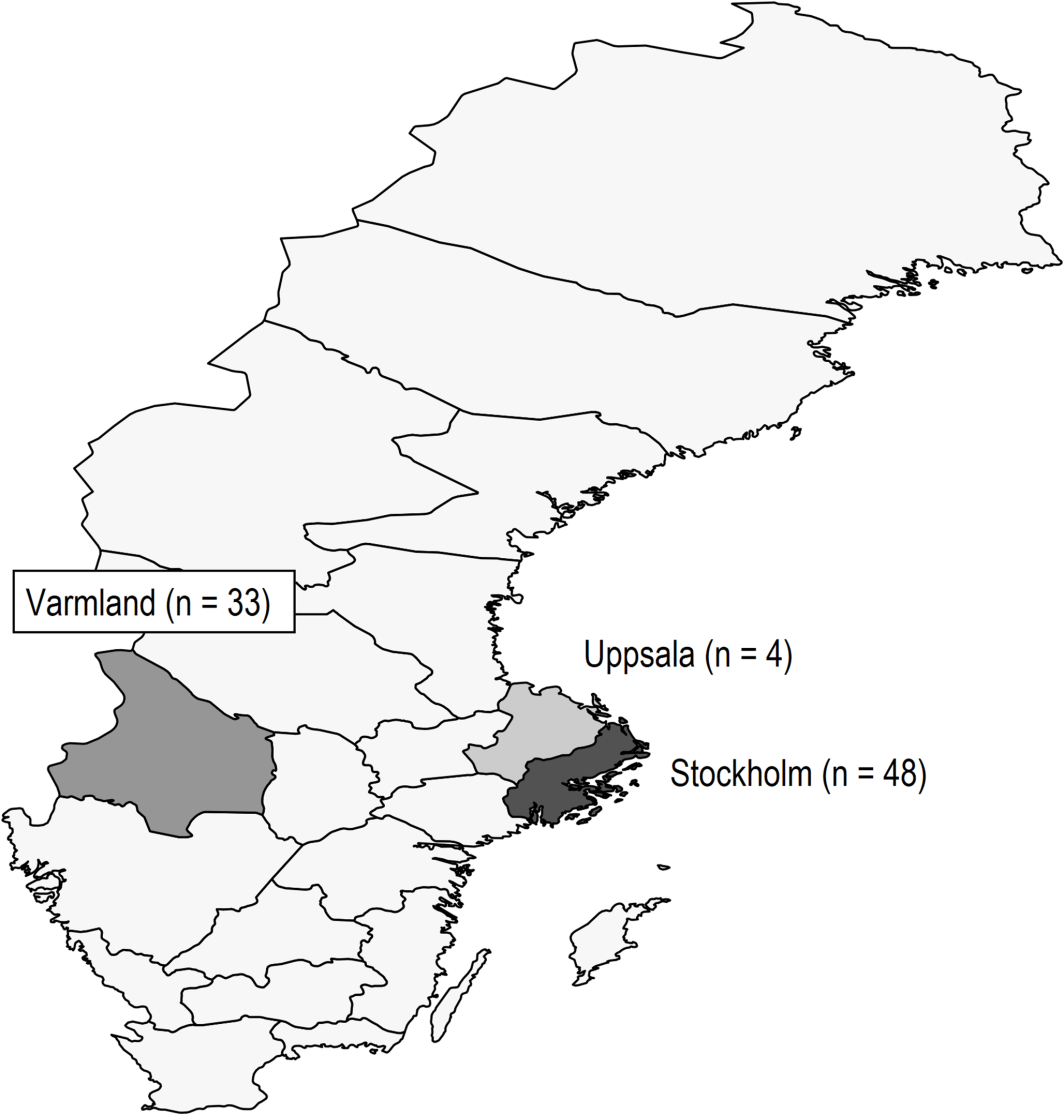
Three regions of Sweden included in the study (n of prehospital whole-blood cases)

Data from Sweden came from three regions of Sweden (Stockholm, Uppsala, and Varmland) that currently institute WB therapy in the field (Fig. 1). Swedish EMS teams initiating WB include a physician. Varmland region is centered in Karlstad, a metropolitan region with a population of around 100,000, located approximately 200 miles to the east of Stockholm. The base responds in a broad area in southern Sweden and occasionally into Norway. Uppsala, with a population nearly 200,000, is located 50 miles north of Stockholm and covers areas north of Stockholm and occasionally the Finnish Aland islands in the Baltic. The Stockholm region has one WB-initiating ground vehicle that covers 2.4 million inhabitants in an area of 2500 square miles. Ground response and initiation of care may be followed by transport to the hospital by either helicopter or ground ambulance [5].

**Fig. 2:**
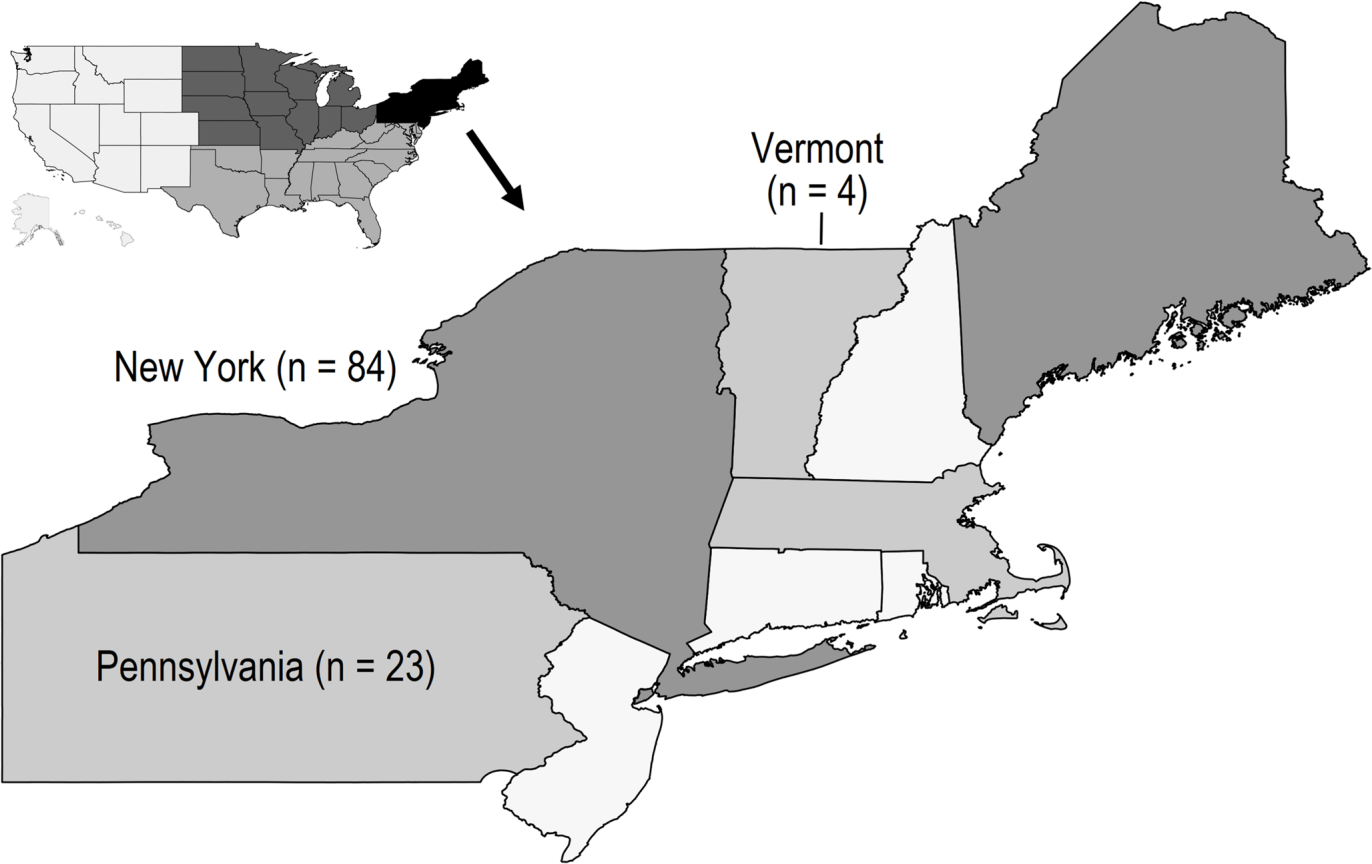
United States Northeast region (n of prehospital whole-blood cases)

Data from the USA were drawn from several Helicopter EMS (HEMS) providers operating in the Northeast (New York, Pennsylvania, and Vermont; see Fig. 2). These three states have a combined population of approximately 33 million.

Study information from both Sweden and USA was drawn from prehospital medical records sources. Ethics approval was obtained from both Sweden and USA authorities.

### Patients

Patients were included if they received WB initiated in the prehospital setting. US cases included all patients from January–September 2024. Sweden had fewer WB cases, so we included five years of data (2020–2024). We did not include patients in whom WB transfusion was considered or planned but not commenced.

### Analysis

Data were analyzed using Stata (version 18.5MP, www.stata.com), which was used for all calculations and plotting. Descriptive statistics for categorical data reported proportions with binomial exact 95% CIs. Categorical comparisons were executed using Pearson’s chi-square or Fisher’s exact test. Effect estimates were calculated as risk ratio (RR) with its 95% CI.

Patient age, found to be non-normally distributed by Shapiro-Wilk testing, was described using medians and interquartile ranges (IQR), with between-group comparisons performed using the Wilcoxon rank-sum test. Median differences with 95% CIs were calculated using the Hodges-Lehmann method.

## Results

The study accrued 196 patients, with slightly more from USA (56.6%) than Sweden. Varmland contributed cases covering 2020–2023, Stockholm covering 2022–2023, and Uppsala for the year 2024; all USA cases were from 2024. By study design, all USA cases involved HEMS transports. A majority (*n* = 48, 56.5% of 85) of Sweden cases arose from the Stockholm region for which WB was administered by EMS responding in ground units (Stockholm patients could have been transported from scenes in either ground or helicopter ambulances).

Table 1 provides information on patient characteristics and on comparisons of Sweden vs. USA WB cases. There were few missing data, involving only the variables of age (*n* = 1 missing, 0.5% of 196) and sex (*n* = 11 missing, 5.6% of 196). Of the 11 cases with missing sex, one was age 53 and thus categorizable with respect to (non) FCP status. All missing data were from Sweden, allowing execution of sensitivity analyses (reported in Supplement).

**Table 1:** Patient characteristics: n of 196 patients with prehospital whole blood transfusion

Characteristic	Overall	Sweden	USA	*p*
*n*	196	85 (43.4% of 196)	111 (56.6% of 196)	-
Trauma (%)	157 (80.1% of 196)	77 (90.6% of 85)	80 (72.1% of 111)	0.001
*Age (median, IQR)	53 (32–67)	41 (30–61)	59 (35–73)	0.003
*Sex (% female)	59 (31.9% of 185)	28 (37.8% of 74)	31 (27.9% of 111)	0.157
*Females ≤ 50 (%)	27 (14.5% of 186)	18 (24.0% of 75)	9 (8.1% of 111)	0.003
> 1 WB unit started	37 (18.9% of 196)	21 (24.7% of 85)	16 (14.4% of 111)	0.068
≥ 1 WB unit finished prehospital	148 (75.5% of 196)	67 (78.8% of 85)	81 (73.0% of 111)	0.345
≥ 2 WB unit finished prehospital	28 (14.3% of 196)	19 (22.4% of 85)	9 (8.1% of 111)	0.005
RhD-positive WB initiated (all cases)	116 (59.2% of 196)	8 (9.4% of 85)	108 (97.3% of 111)	< 0.001
RhD-positive WB started in FCP	10 (37.0% of 27)	1 (5.6% of 18)	9 (100% of 9)	< 0.001

 Sweden cases were more likely to have a trauma diagnosis (RR 1.26, 95% CI 1.10-1.44) and a median of 10 (95% CI 3 to 17) years younger than USA cases. Despite similar proportions of all-age females in Sweden and the USA, Sweden WB recipients had a significantly higher likelihood of FCP status (RR 2.96, 95% CI 1.41-6.23). Still, USA FCPs’ were more likely to receive D-positive WB (RR 18, 95% CI 2.68-120).

 While all patients had at least one unit of WB initiated by EMS, Sweden cases had a significantly higher chance of having multiple WB units completed prior to hospital arrival (RR 2.76, 95% CI 1.31-5.78).

## Discussion

 We found marked differences in prehospital WB recipients between Sweden and the USA Northeast. These differences are clinically meaningful and should be considered when developing transfusion strategies or evaluating their impact. This study does not assess whether one region’s approach is superior, but highlights variation that may affect generalizability of findings or modeling assumptions.

 First, we found that overall, one in five WB administrations was for an indication other than trauma. High-level evidence guiding civilian WB use remains limited [3] [6] but there are more data informing WB benefits for trauma than for other diagnoses. Sweden prehospital WB patients were also a decade younger than those in the USA and were nearly three times as likely to complete multiple WB units before hospital arrival.

We also considered risks, focusing on alloimmunization. Hemolysis and related complications are possible in any RhD-negative patient receiving RhD-positive WB[7], Our findings indicated that RhD-positive WB was received by 100% of USA FCPs as compared to 6% of Sweden FCPs; the potential risks and costs of prehospital WB should be considered different in the two studied populations. This only implies higher *potential* alloimmunization risk in the U.S. group; we did not have follow-up data on anti-RhD antibody development or RhD immunoprophylaxis.

##  Limitations

 We studied only three of the four Swedish WB regions and one U.S. Northeast region. The unsampled Swedish region (Västra Götaland) or other U.S. areas might have different patient profiles or practices, so our findings may not be nationally representative. We lacked certain data elements: we did not have hospital outcomes (e.g. survival), and we did not have patients’ actual RhD status beyond the transfused units. Importantly, none of the datasets included follow-up antibody testing or information on Rh immunoprophylaxis. We also did not have access to all applicable protocols of participating EMS systems, but the indications for WB were not the focus of the current study.

An additional limitation of the current study is that transport times were not reliably available. Given recent evidence that time savings translate to improved outcome with prehospital transfusion[1], and that prehospital transfusion may be most useful when at least 20 minutes are saved[8], future studies of EMS WB administration should also evaluate time saved with prehospital initiation of therapy.

 We also lacked sufficient numbers of FCP cases to generate highly precise estimates of their proportions (but there were sufficient FCPs to detect the Sweden-USA difference as statistically significant).

##  Conclusion

 In summary, compared to the U.S. Northeast, Swedish WB recipients were younger, more often trauma patients, and more often female of childbearing potential. Swedish EMS transfused multiple units more frequently, and U.S. patients were more likely to receive RhD-positive blood. These differences highlight the need to tailor WB analysis to local context. Models should not assume demographic or procedural equivalence across regions.

## Supplementary Information

Below is the link to the electronic supplementary material.

Supplementary Material 1 (DOCX 20.4 KB)

## Data Availability

No datasets were generated or analysed during the current study.
