# Supplementary material for: Prehospital whole-blood transfusion in two countries: comparison of patient characteristics in Sweden and the Northeastern United States
Source: Eur J Trauma Emerg Surg. 2026 Jan 13;52(1):19. doi: 10.1007/s00068-025-03037-9 (PMC12799669; doi:10.1007/s00068-025-03037-9)
Supplement: Supplementary file 1 — Supplementary Material 1 (DOCX 20.4 KB) [file 68_2025_3037_MOESM1_ESM.docx]

**Supplement**

The supplemental information provided here is intended to complement the main-paper information on methodology and results. Supplemental topics are as follows:

*S1: Ethics approval and STROBE checklist*

*S2: Sensitivity analysis (assessing effect of missing demographic data)*

*S1: Ethics approval and STROBE checklist*

The study was approved at the USA base site institutional review board (#2023P001106). Additional approval was obtained from the Swedish Etikprövningsmyndigheten (#2024-02270-01-592049).

Appendix Table 1 provides STROBE checklist information.^4^

Appendix Table 1. STROBE checklist

|  | Item No | Recommendation [*location in manuscript*] |
| --- | --- | --- |
| **Title and abstract** | 1 | (*a*) Indicate the study’s design with a commonly used term in the title or the abstract [*Abstract*] |
|  |  | (*b*) Provide in the abstract an informative and balanced summary of what was done and what was found [*Abstract*] |
| Introduction | | |
| Background/rationale | 2 | Explain the scientific background and rationale for the investigation being reported [*Background*] |
| Objectives | 3 | State specific objectives, including any prespecified hypotheses [*Background*] |
| Methods | | |
| Study design | 4 | Present key elements of study design early in the paper [*Methods*] |
| Setting | 5 | Describe the setting, locations, and relevant dates, including periods of recruitment, exposure, follow-up, and data collection [*Methods*] |
| Participants | 6 | (*a*) Give the eligibility criteria, and the sources and methods of selection of participants. Describe methods of follow-up [*Methods*] |
|  |  | (*b*) For matched studies, give matching criteria and number of exposed and unexposed [*Not applicable*] |
| Variables | 7 | Clearly define all outcomes, exposures, predictors, potential confounders, and effect modifiers. Give diagnostic criteria, if applicable [*Methods*] |
| Data sources/ measurement | 8 | For each variable of interest, give sources of data and details of methods of assessment (measurement). Describe comparability of assessment methods if there is more than one group [*Methods*] |
| Bias | 9 | Describe any efforts to address potential sources of bias [*Methods & Supplement*] |
| Study size | 10 | Explain how the study size was arrived at [*Methods*] |
| Quantitative variables | 11 | Explain how quantitative variables were handled in the analyses. If applicable, describe which groupings were chosen and why [*Methods*] |
| Statistical methods | 12 | (*a*) Describe all statistical methods, including those used to control for confounding [*Methods*] |
|  |  | (*b*) Describe any methods used to examine subgroups and interactions [*Methods*] |
|  |  | (*c*) Explain how missing data were addressed [*Methods & Supplement*] |
|  |  | (*d*) If applicable, explain how loss to follow-up was addressed [*Supplement*] |
|  |  | (*e*) Describe any sensitivity analyses [*Supplement*] |
| Results | | |
| Participants | 13 | (a) Report numbers of individuals at each stage of study—eg numbers potentially eligible, examined for eligibility, confirmed eligible, included in the study, completing follow-up, and analysed [*Figure 1*] |
|  |  | (b) Give reasons for non-participation at each stage [*Not applicable*] |
|  |  | (c) Consider use of a flow diagram [*Figure 1*] |
| Descriptive data | 14 | (a) Give characteristics of study participants (eg demographic, clinical, social) and information on exposures and potential confounders [*Results & Supplement*] |
|  |  | (b) Indicate number of participants with missing data for each variable of interest [*Figure 1*] |
|  |  | (c) Summarise follow-up time (eg, average and total amount) [*Methods*] |
| Outcome data | 15 | Report numbers of outcome events or summary measures over time [*Results & Supplement*] |
| Main results | 16 | (*a*) Give unadjusted estimates and, if applicable, confounder-adjusted estimates and their precision (eg, 95% confidence interval). Make clear which confounders were adjusted for and why they were included [*Results & Supplement*] |
|  |  | (*b*) Report category boundaries when continuous variables were categorized [*Methods*] |
|  |  | (*c*) If relevant, consider translating estimates of relative risk into absolute risk for a meaningful time period [*Not applicable*] |
| Other analyses | 17 | Report other analyses done—eg analyses of subgroups and interactions, and sensitivity analyses [*Supplement*] |
| Discussion | | |
| Key results | 18 | Summarise key results with reference to study objectives [*Results & Supplement*] |
| Limitations | 19 | Discuss limitations of the study, taking into account sources of potential bias or imprecision. Discuss both direction and magnitude of any potential bias [*Discussion*] |
| Interpretation | 20 | Give a cautious overall interpretation of results considering objectives, limitations, multiplicity of analyses, results from similar studies, and other relevant evidence [*Discussion*] |
| Generalisability | 21 | Discuss the generalisability (external validity) of the study results [*Discussion*] |
| Other information | | |
| Funding | 22 | Give the source of funding and the role of the funders for the present study and, if applicable, for the original study on which the present article is based [*Title page – study conducted using only internal funding from lead investigators’ institution*] |

*S2: Sensitivity analysis – potential effect of missing data on Sweden* vs. *USA demographic comparisons*

As noted in the main results, all of the missing data were from the Sweden cohort. This enabled a sensitivity analysis that replaced all missing-data values with extreme values to determine if the main results changed.

For age, there was only one missing value. If this missing value was replaced with either the youngest observed age (4) or the oldest observed age (93), the significant difference between ages remained (*p* < .01 for each sensitivity analysis). The estimated median difference between Sweden and USA patients’ ages remained 10 (95% CI 4-17) when the missing age value was substituted with age 4; the median inter-country age difference was 9 (95% CI 3-16) when the missing value was substituted with age 93.

For sex, there were two sets of sensitivity analyses. The first focused solely on whether the observed lack of difference between Sweden and USA proportion of females remained when missing-sex (Sweden) cases were all coded as male (first analysis) or all were coded as female (second analysis). If the overall proportion of females (37.8%) was applied to the missing 11 cases, there remained no association (*p* = .149) between country and proportion of females. Similarly, when all Sweden missing-sex cases were coded as male, there remained no difference (*p* = .594) between Sweden and USA proportions of females. When as many as eight of the missing 11 cases (73%) were coded as female, the difference between Sweden and USA proportion of females became significant (*p* = .035).

The FCP calculations’ sensitivity analysis was based on the missing FCP status in *n* = 10 cases. Even if all 10 of the missing-FCP cases were not FCP, the Sweden WB recipients would still have a significantly (*p* = .009) higher proportion of FCP than USA WB recipients.
